# Efficacy and complications of single-port thoracoscopic minimally invasive esophagectomy in esophageal squamous cell carcinoma: a single-center experience

**DOI:** 10.1038/s41598-023-41772-4

**Published:** 2023-09-28

**Authors:** Fei Zheng, Jun Yang, Jiulong Zhang, Jiancheng Li, Weimin Fang, Mingqiu Chen

**Affiliations:** 1 Department of Radiation Oncology, Clinical Oncology School of Fujian Medical University, Fujian Cancer Hospital, No. 420 Fuma Rd. Jin’an District, Fuzhou, 350014 Fujian Province People’s Republic of China; 2 Department of Thoracic Surgery, Clinical Oncology School of Fujian Medical University, Fujian Cancer Hospital, No. 420 Fuma Rd. Jin’an District, Fuzhou, 350014 Fujian Province People’s Republic of China

**Keywords:** Diseases, Medical research, Oncology

## Abstract

The traditional surgical technique for esophageal cancer is mainly open esophagectomy. With the innovation of surgical instruments, it is necessary to re-optimize the minimally invasive surgery. Therefore, single-port thoracoscopic minimally invasive esophagectomy (SPTE) is an important direction of development. This study retrospectively analyzed 202 patients with esophageal squamous cell carcinoma undergoing SPTE. Surgical variables and postoperative complications were further evaluated. All procedures were performed using SPTE. The number of patients who received R0 resection was 201 (99.5%). The total number of resected lymph nodes during the whole operation was on average 32.01 ± 12.15, and the mean number of positive lymph nodes was 1.56 ± 2.51. In 170 cases (84.2%), intraoperative blood loss did not exceed 100 ml (ml), while 1 case had postoperative bleeding. Only 1 patient (0.5%) required reoperation after surgery. Postoperative complications included 42 cases of pneumonia (20.8%), 9 cases of anastomotic leak (4.5%), 7 cases of pleural effusion (3.8%), and 1 case (0.5%) of both pleural hemorrhage and acute gastrointestinal hemorrhagic ulcer. Besides, we also recorded the time to remove the drain tube, which averaged 9.13 ± 5.31 days. In our study, we confirmed that the application of SPTE in clinical practice is feasible, and that the postoperative complications are at a low level.

## Introduction

According to global cancer statistics, esophageal cancer causes more than 540,000 deaths each year, ranks sixth among the top 10 cancers and accounts for 5.56% of all cancer deaths. There are gender differences in the incidence of esophageal cancer, with men accounting for about three-quarters of the population. It is mainly due to bad lifestyle and eating habits^1–3^. Surgery performs an essential position in the treatment of esophageal cancer, mainly patients in the early stages, and is recognized as the gold standard of treatment^4^.

Traditional surgical techniques for esophageal cancer are mostly open esophagectomy (OE). The various central surgical techniques are still controversial. Depending on the location of the tumor and the status of the lymph nodes, the most commonly used surgical methods are cervical esophago-gastrostomy, transthoracic subtotal esophagectomy with intrathoracic and Sweet’s left thoracoabdominal approach, and transhiatal extended gastrectomy^5,6^. Considering the anatomical complexity of the esophagus and the variety of lymphatic drainage, from a normative point of view, in the case of middle and lower thoracic esophageal cancer, the dissection of two or three field lymph nodes in the cervical, mediastinal, and abdominal is required^7^. However, traditional thoracotomy can be fatal in patients who cannot tolerate it^8^. Thus, with the advent of minimally invasive surgery, these obstacles have been addressed to some extent. The development of minimally invasive esophagectomy (MIE) dates back to the 1990s, when Cuschieri and colleagues first reported the use of the MIE technique in clinical practice^9^. Subsequent explorations used a variety of surgical positions, including left lateral decubitus, prone and modified semi prone position^10–12^. However, complete lung collapse is required during the procedure, which greatly increases the incidence of pulmonary complications^10^.

In recent years, with the modernization of surgical instruments and the emphasis on perioperative management, especially the concept of rapid recovery after surgery, there is a need to re-optimize MIE. Single-port thoracoscopic minimally invasive esophagectomy (SPTE) is an important direction in the development of MIE. Some centers have performed preliminary studies in this field, all patients achieved R0 resection, and the median number of lymph nodes resected was 16. Also, there were no postoperative deaths, serious postoperative complications, such as anastomotic leakage or conversion to minimally invasive surgery^13^. However, due to the limited number of samples, the clinical utility of this technique is still controversial. In this study, 202 patients with SPTE were collected and retrospectively analyzed for final surgical efficacy and postoperative complications to provide a reference for the development and optimization of single-port surgery.

## Methods

### Patients

We retrospectively analyzed 202 patients from July 1, 2018 to February 28, 2022 with esophageal squamous cell carcinoma who receiving SPTE. This study was approved by the Ethics Committee of Fujian Cancer Hospital. all methods were performed in accordance with the relevant guidelines and regulations. Written informed consent was obtained from all patients and information was anonymized prior to analysis. Inclusion criteria: 1. Esophageal squamous cell carcinoma confirmed by histopathology; 2. In patients without distant metastases, tumor stage was determined according to the American Joint Committee on Cancer (AJCC) 7th edition staging system. 3. Eastern Cooperative Oncology Group performance status ≤ 2; 4. Patients and their family members accept surgical treatment. Exclusion criteria: 1. Suffering from severe liver and kidney disorders; 2. Distant metastases confirmed before surgery; 3. History of other tumors. Neoadjuvant chemotherapy was a double drug regimen consisting of platinum-based + paclitaxel/docetaxel. Intensity modulated radiation therapy (IMRT) was used in neoadjuvant radiotherapy with a total dose of 45–62 Gy and a fractional dose of 1.8–2.0 Gy.

### Thoracic phase

#### Position

**Figure 1:**
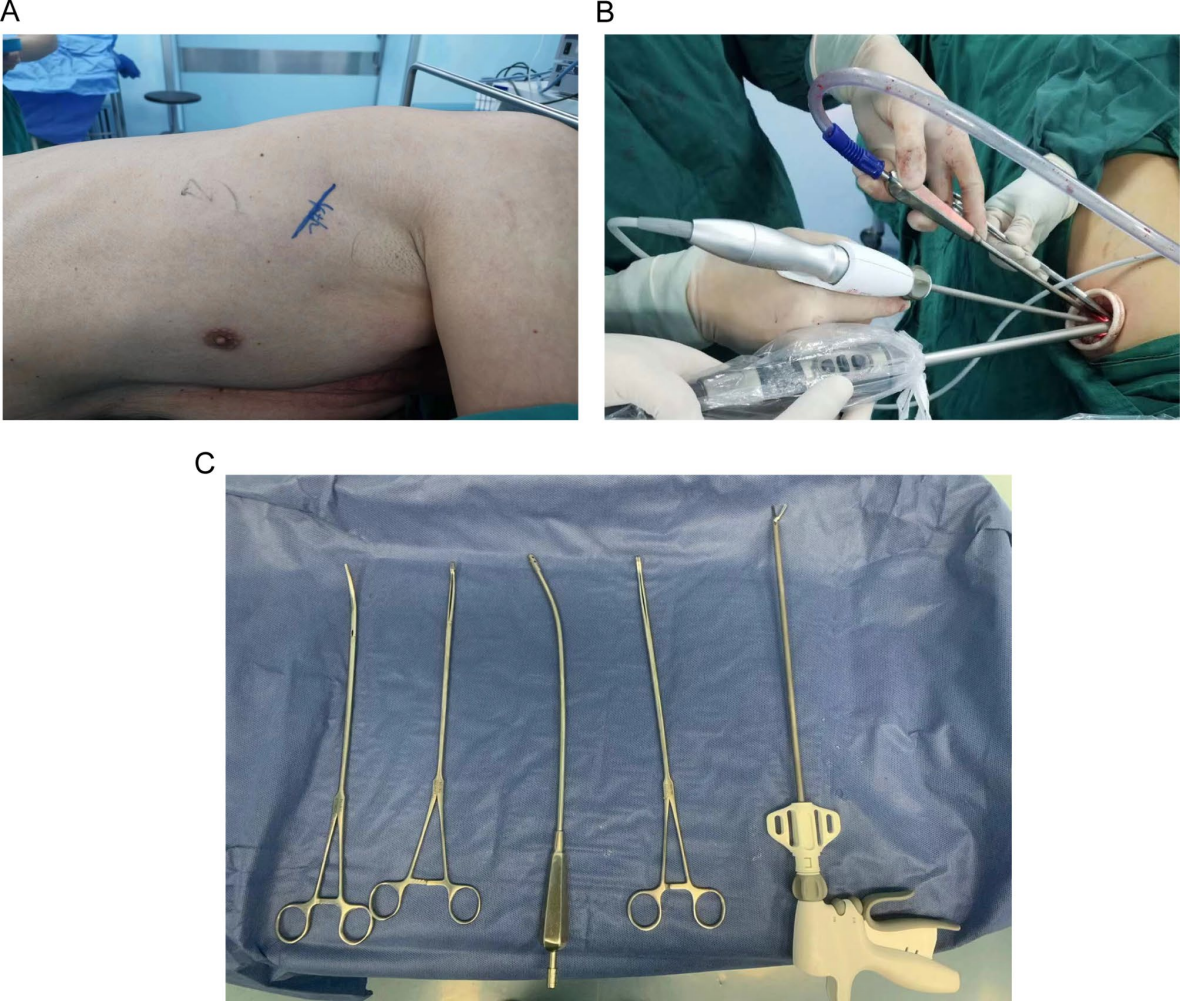
Single-port thoracoscopic minimally invasive esophagectomy in patients with esophageal squamous cell carcinoma. (**A**) The left lateral prone position was adopted, and the surgical incision was selected as the fourth intercostal of the posterior axillary line, with an incision length of 3 cm. (**B**) Position of instruments and operators in thoracic phase. (**C**) Special instruments used during the operation. From left to right: double-joint vascular forceps for separating tissue; double-joint snakehead forceps exposing surgical field or transporting gauze; elbow suction apparatus for sucking blood and smoke; double-joint ring forceps for immobilizing soft tissues or exposing surgical field; ultrasonic harmonic scalpel for separating tissue or stopping bleeding.

Under general anesthesia and double-lumen intubation, the patient was placed in a left lateral position (90°) (Fig. 1A). The three participants in the procedure were positioned as follows: the operator and endoscopist were on the patient's left side, while the assistant was on the right side. Locate the surgical incision on the posterior axillary line of the 4th intercostal space of the right chest wall and made a 3 cm incision. Then insert a wound protector into the incision. To obtain a clear view of the operation, the camera was inserted into a 12 mm cannula, positioned between the wound and wound protector (Fig. 1B).

#### Mobilization of the esophagus and lymph node dissection in the mediastinal region

**Figure 2:**
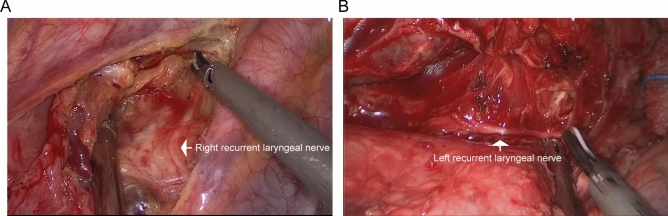
Lymph node dissection at the thoracic period. (**A**) Lymph node dissection along the right recurrent laryngeal nerve (white arrow). (**B**) Lymph node dissection along the left recurrent laryngeal nerve (white arrow).

Thoroughly investigated the tumor to determine the extent of adhesion to surrounding tissues and the presence of pleural effusion. The pleura was separated along the lower edge of azygos vein. Under endoscopic view, we performed dissection and segmentation of the azygos arch using a linear stapler. We used a conventional hook cautery and harmonic ultrasound scalpel to free the space between the esophagus and surrounding tissue or lymph nodes, up to the azygos arch to the subclavian artery level, and to provide good exposure to the right recurrent nerve, then down to the esophageal hiatus of the diaphragm. For the dissection of mediastinal lymph nodes, it mainly included the lymph nodes below the carina and the bilateral recurrent laryngeal nerve regions (Fig. 2A, B). During single port surgery, the dual-joint tongue forceps were used to assist in esophageal retraction (Fig. 1C). Not only can it firmly traction the esophagus, but it also won't cause any damage to the esophagus tissue. After the chest surgery, a 28F chest tube was inserted through the incision in the chest wall, followed by suturing of the incision. An elbow suction apparatus can be used to remove blood, fluid, and smoke, as well as to assist in exposing the surgical field of view (Fig. 1C).

### Abdominal and cervical phase

Patients were placed in a supine position and the area re-sterilized. The mobilization of the esophagus in the abdominal segment and stomach with celiac lymph node dissection were achieved through laparoscopic approach based on the technique of five ports technique. Subsequently, we made a 3 cm incision in the upper mid-abdominal of the patient, primarily for the formation of an extracorporeal stomach conduit and removal of specimens. Four threads were inserted through the serous and muscular layers at the top of the gastric tube to serve as traction. We pulled four threads to guide the gastric tube from the posterior mediastinum through the esophageal bed to the left side of the neck. A double-layered anastomosis was created for the cervical area with the help of a circular stapler. After anastomosis was achieved, insert nasogastric tube. After inspecting the neck and abdominal cavity individually and not observing any active bleeding, proceed to suture the abdominal and neck incisions layer by layer.

### Outcome variable

Perioperative outcomes of all cases were summarized and observed data included: node counts, resection R0/R1/R2, bleeding during surgery, postoperative bleeding, reoperation after surgery, pneumonia, anastomotic leakage, acute gastrointestinal hemorrhagic ulcer, thoracic hemorrhage and pleural effusion, time to remove the drain tube and the length of hospital stay.

## Results

Among 202 patients with esophageal squamous cell carcinoma, 148 (73.3%) male and 54 (26.7%) female, the mean age was 61.9 ± 8.07 years. When divided by tumor location, there were 38 cases (18.8%), 74 cases (36.6%), and 90 cases (44.6%) in the upper, middle, and lower thoracic, respectively. Moreover, we also characterized the length of the tumors with a mean length of 4.38 ± 1.5 cm. Some patients with T2, T3, T4 lesions and positive regional lymph nodes received neoadjuvant chemotherapy, which accounted for 24.8% (50 cases). According to the eighth edition of American Joint Committee on Cancer, T1/2 stage was 78 cases (38.6%), T3/4 was 124 cases (61.4%); N0 was 109 cases (54.0%), N1/2/3 was 93 cases (46.0%) (Table 1).

**Table 1:** Clinical characteristics of patients.

Characteristic	Classify	Overall
n		202
Gender, n (%)	Female	54 (26.7%)
	Male	148 (73.3%)
Age, mean ± SD		61.92 ± 8.07
Length (cm), mean ± SD		4.38 ± 1.5
Location, n (%)	Upper thoracic	38 (18.8%)
	Middle thoracic	74 (36.6%)
	Lower thoracic	90 (44.6%)
Neoadjuvant therapy, n (%)	No	152 (75.2%)
	Yes	50 (24.8%)
cT, n (%)	1	45 (22.3%)
	2	33 (16.3%)
	3	103 (51%)
	4	21 (10.4%)
cN, n (%)	0	109 (54%)
	1	48 (23.8%)
	2	34 (16.8%)
	3	11 (5.4%)

*c* clinical staging, *T* tumor, *N* node, *SD* standard deviation.

All operations were performed using SPTE without conversion to multiport thoracoscopy or OE and were shown in Table 2. In the pathological stage of patients, T1/2 was 85 cases (42.1%), T3/4 in 117 cases (57.9%), N0 in 113 cases (55.9%), and N1/2/3 in 89 cases (44.1%). The number of patients who undergoing R0 resection was 201 (99.5%), R1 in 1 (0.5%) and R2 in 0 cases. The total number of resected lymph nodes during the whole operation was on average 32.01 ± 12.15, and the mean number of positive lymph nodes was 1.56 ± 2.51. In 170 cases (84.2%), intraoperative blood loss did not exceed 100 ml, while 1 case had postoperative bleeding. It is worth mentioning that no postoperative bleeding was observed in all patients. Only 1 patient (0.5%) required reoperation after surgery. Postoperative complications included 42 cases of pneumonia (20.8%), 9 cases of anastomotic leak (4.5%), 7 cases of pleural effusion (3.8%), and 1 case of both pleural hemorrhage and acute gastrointestinal hemorrhagic ulcer (0.5%). At the same time, we also recorded the time to remove the drain tube, which averaged 9.13 ± 5.31 days. In addition, the mean length of hospital stay for all patients was 20.72 ± 8.23 days.

**Table 2:** Outcomes of perioperative.

Characteristic	Classify	Overall
n		202
Resection, n (%)	R0	201 (99.5%)
	R1	1 (0.5%)
	R2	0 (0%)
Node counts, mean ± SD		32.01 ± 12.15
Positive nodes, mean ± SD		1.56 ± 2.51
pT, n (%)	1	48 (23.8%)
	2	37 (18.3%)
	3	106 (52.5%)
	4	11 (5.4%)
pN, n (%)	0	113 (55.9%)
	1	45 (22.3%)
	2	33 (16.3%)
	3	11 (5.4%)
Bleeding during surgery (ml), n (%)	≤ 100	170 (84.2%)
	> 100	32 (15.8%)
Postoperative bleeding (ml), n (%)	0	202 (100%)
	> 0	0 (0%)
Reoperation after surgery, n (%)	No	201 (99.5%)
	Yes	1 (0.5%)
Pneumonia, n (%)	No	160 (79.2%)
	Yes	42 (20.8%)
Anastomotic leakage, n (%)	No	193 (95.5%)
	Yes	9 (4.5%)
Other postoperative complications, n (%)	Acute gastrointestinal hemorrhagic ulcer	1 (0.5%)
	Thoracic hemorrhage	1 (0.5%)
	Pleural effusion	7 (3.8%)
Time to remove the drain tube (day), mean ± SD		9.13 ± 5.31
Length of hospital stay		20.72 ± 8.23

*p* pathological staging, *T* tumor, *N* node, *SD* standard deviation.

## Discussion

Surgery has been used as the fundamental treatment of esophageal cancer. Patients initially undergoing surgery for esophageal cancer most of them are thoracotomy. However, traditional open surgery still has limitations. For one thing, relatively large lesions cause a slow recovery after surgery, which greatly affects the quality of life of patients. For another, since open surgery requires a very high physiological state of the patient, there are many patients who lose the possibility of long-term survival because poor physical strength that prevents them from undergoing basic surgery. Although some studies suggest that in patients with esophageal cancer that cannot be surgically resected due to physiologic reasons or limitations of tumor localization, radical concurrent chemoradiotherapy may be the optimal choice and achieve the same effect as surgery^14^. Many meta-analyses have shown that surgery still plays an important role in the treatment of tumors compared to concurrent chemoradiotherapy^15,16^. This is attributed to the fact that surgery not only largely removes the primary tumor and reduces the tumor burden, but also reduces the risk of distant metastases, which is difficult to achieve with concurrent chemoradiotherapy. Therefore, surgery remains the best treatment for esophageal cancer.

Recently, as MIS has made significant progress, age is no longer a contraindication for esophageal cancer surgery. Therefore, the population of surgery benefits is further expanded. At the same time, the cost of training time for the chief surgeon has been significantly reduced, which has overcome the long waiting times caused by the increase in surgical populations. Technological innovation provides a win–win situation. However, the current rate of postoperative pulmonary complications is still 8–33.9% due to artificial pneumothorax intervention during MIS^17–21^. Especially in elderly patients, this may lead to a direct reduction in survival^22^, which should also be taken into account by the clinical medical workers.

Single-port thoracoscopic surgery is favored as an innovative and minimally invasive technique for thoracic surgery. It minimizes interference with the patient's normal physiological functions during surgery while preserving the integrity of the surrounding tissues to the greatest extent possible, leading to a reduced risk of vascular injury and bleeding. The significant reduction in operative time also means shorter anesthesia time, which in turn reduces the burden on the patient's body and the risk of complications. Additionally, patients experience better cosmetic results due to the small trauma and the lack of need for selective wound closure^23^. Patients often experience rapid relief of surgery-related pain and discomfort after surgery, and their level of early recovery is significantly improved, resulting in a quicker return to normal life and work^20^. Therefore, the less invasive SPTE has become an important technique pursued by numerous surgeons. Although less invasive, SPTE is also an extremely complex and challenging operation. Also, its postoperative complications should be taken seriously. Previous studies have confirmed that the postoperative complications of MIE are much lower than those of OE^24–27^. We retrospectively analyzed SPTE and found that the overall postoperative complication rate was 29.7%, which was significantly lower than 80.0–86.6% for OE and 55.3–59.0% for MIE^20,28^. However, given the single-center study and small number of cases, its results should be evaluated rationally. A meta-analysis of 16,053 patients showed an overall complication rate of 45% for OE and 37% for MIE^29^. In addition, patients may benefit from MIE in terms of long-term survival. A multicenter study from Europe showed that 3-year OS was 50.5% in the MIE group and 40.4% in the OE^30^. To some extent, reducing trauma and postoperative complication can significantly extend the long-term survival of patients. And this can only be achieved by changing the surgical method.

In our study, the highest incidence of pneumonia among all postoperative complications was observed and reached 20.8%. In MIE, the rate of postoperative pulmonary complications was 16–32.0%, and 44.0–58.0% in OE^20,31–33^. The incidence of pleural effusion was 3.8%, which was similar to previously reported MIE of 3.7% and OE of 5.5%^20^. Besides, 1 case (0.5%) developed acute gastrointestinal hemorrhagic ulcer. This complication is thought to be related to surgical stress and can be prevented or made less severe with medication. It is well known that anastomotic leakage is one of the more serious complications occurring after esophagectomy. It not only directly leads to prolonged hospital stay, but also increases the burden of medical costs^34^. More importantly, it brings serious impact on the prognosis and quality of life of patients^35,36^. Because of the long-term physical discomfort faced by the patients, the psychological pressure increases, the mood is depressed, and even causes psychological disorders, which further affects the socialization ability. In addition, due to the long-term inadequate nutritional intake will make the patient's physical strength decline, resistance is weakened, and it is easy to be attacked by infectious diseases. All these factors can threaten the long-term survival of patients and should receive more attention. In addition, our study showed that the anastomotic leakage rate was 4.5%, which was significantly lower than the incidence of anastomotic fistula in MIE ranging from 6.1 to 14.4%^37,38^. Further analysis showed that anastomotic leakage occurred most frequently in elderly and malnourished patients, as a major factor affecting anastomotic healing. This also illustrates the obvious advantage of SPTE in reducing anastomosis leakage complications.

Blood plays a central role in body homeostasis, and reducing intraoperative bleeding is a main objective for surgeons. Surgical trauma is directly proportional to the amount of bleeding, so technological innovations will greatly contribute to the development of this field. Compared with 374.38 ± 415.34 ml in conventional OE, blood loss in MIE was significantly reduced to 209.59 ± 169.02 ml^27^. In this study, we also evaluated bleeding during SPTE. Bleeding volume less than or equal to 100 ml accounted for 84.2% of the total population, with the largest bleed being 900 ml and only one person. This means that fewer patients require intraoperative blood transfusion support, which is an independent factor in long-term survival^39^.

When resecting esophageal cancer, regional lymph nodes should be considered as the first stop for the spread of esophageal cancer. Previous studies have shown that the number of resected regional lymph nodes significantly correlates with OS and PFS in patients^40^. Studies showed that patients who had ≥ 15 lymph nodes removed had an improved median OS compared with those who had < 15 lymph nodes removed, 42.0 versus 37.1 months, respectively. As expected, when ≥ 25 lymph nodes were harvested, OS was further improved to 55.4 months^41^. In our study, the mean number of lymph nodes sampled was 32, and the mean number of positive lymph nodes was 1.56. It is precisely because of the use of MIE that three-dimensional visualization of the surgical area can be realized. At the same time, surgical precision can be further improved due to greater mobility of the surgical instrument and magnification effect of view.

## Conclusion

SPTE is a feasible and valuable ultra-minimally invasive operation, but requires more accurate preoperative staging, stricter indications, and more skilled multiport thoracoscopy. However, as this is a retrospective study, bias is inevitable. Therefore, a prospective randomized controlled trial to confirm the efficacy and safety of SPTE is a direction worth exploring. In fact, the gold standard for the assessment of postoperative complications is the Esophagectomy Complications Consensus Group (ECCG) guidelines. This study only listed the major, high incidence and potentially life-threatening complications is one of the other limitations of the study. However, the complications involved in this study are also important references for subsequent studies of SPTE. In addition, SPTE is more complex to perform and is recommended for experienced and well-trained surgeons.

## Data Availability

The datasets used the current study are available from the corresponding author (CM) on reasonable request.
